# Supplementary material for: The Developing Human Connectome Project: typical and disrupted perinatal functional connectivity
Source: Brain. Author manuscript; Available in PMC 2021 Aug 24. (PMC8370420; doi:10.1093/brain/awab118)
Supplement: Supplementary Material [file EMS123325-supplement-Supplementary_Material.pdf]

Supplementary Material

The Developing Human Connectome Project: typical and disrupted perinatal functional connectivity

Michael Eyre[a], Sean P Fitzgibbon[b], Judit Ciarrusta[a,c], Lucilio Cordero-Grande[a], Anthony N Price[a], Tanya Poppe[a], Andreas Schuh[d], Emer Hughes[a], Camilla O'Keeffe[a], Jakki Brandon[a], Daniel Cromb[a], Katy Vecchiato[a,c], Jesper Andersson[b], Eugene P Duff[b,e], Serena J Counsell[a], Stephen M Smith[b], Daniel Rueckert[d], Joseph V Hajnal[a], Tomoki Arichi[a,f], Jonathan O'Muircheartaigh[a,c], Dafnis Batalle[a,c†*], A David Edwards[a†*]

[a] Centre for the Developing Brain, School of Biomedical Engineering & Imaging Sciences, King's College London, London, UK
[b] Wellcome Centre for Integrative Neuroimaging (WIN FMRIB), University of Oxford, Oxford, UK
[c] Department of Forensic and Neurodevelopmental Science, Institute of Psychiatry, Psychology and Neuroscience, King's College London, London, UK
[d] Biomedical Image Analysis Group, Imperial College London, London, UK
[e] Department of Paediatrics, University of Oxford, Oxford, UK
[f] Department of Bioengineering, Imperial College London, London, UK

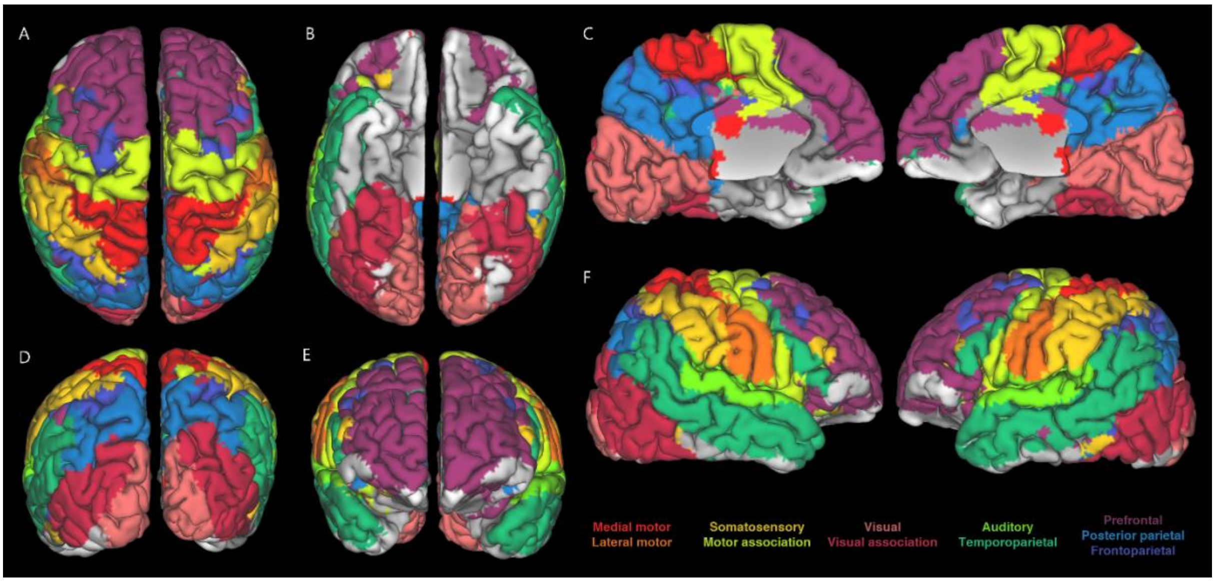

Supplementary Figure S1. Spontaneous BOLD activity patterns (RSNs) derived from group ICA in 24 term-born infants scanned at 43.5-44.5 weeks PMA. RSN are expressed as a functional parcellation of the brain using a 'winner-takes-all' approach based on the RSNs from group ICA. RSNs were spatially smoothed and thresholded at Z > 1 prior to determination of the 'winning' RSN at each voxel. The resulting volume was projected to the midthickness cortical surface using enclosed (nearest neighbour) volume-to-surface mapping, here displayed on the pial surface of an individual subject scanned at 42 weeks PMA and viewed from the superior (A), inferior (B), medial (C), lateral (D), anterior (D) and posterior (E) aspects.

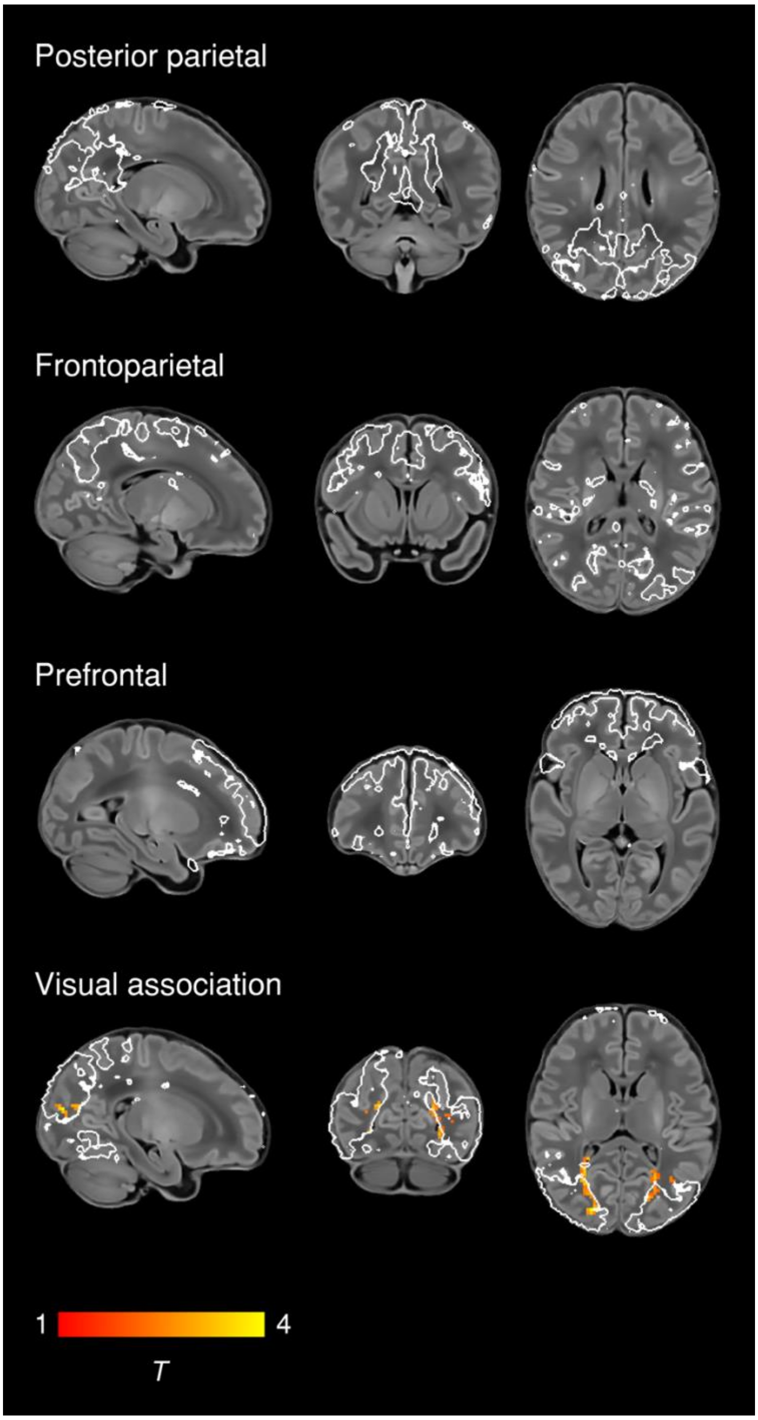

Supplementary Figure S2. Changes in network shape with increasing age at scan (Bonferroni-corrected). Brain regions showing increased functional connectivity with older PMA at scan in term-born infants scanned at 37-43.5 weeks PMA. Example sagittal, coronal, and axial slices for meaningful spatial patterns in four RSNs are shown, overlaid on a T1 structural template and displayed in radiological convention. T-statistic maps were thresholded at $p < 0.025/11$ (i.e. $p < 0.002$) (FWE corrected). White lines represent the outlines of the group-ICA RSNs, thresholded at $Z > 3$.

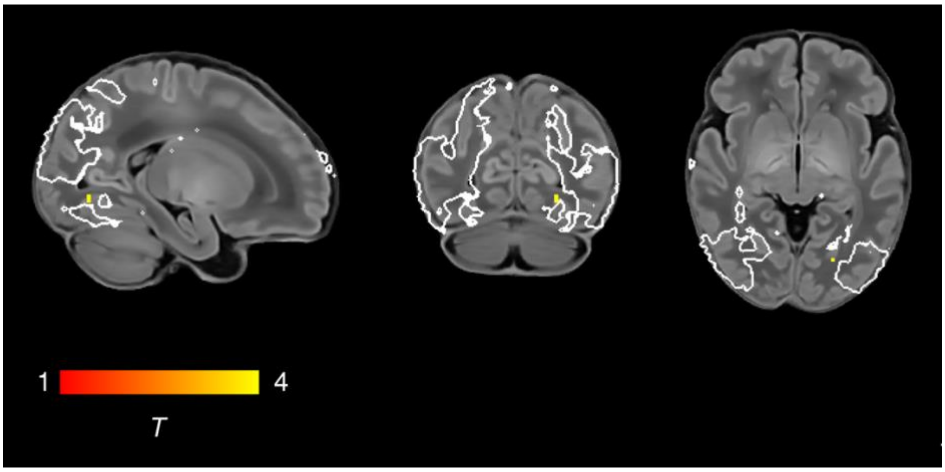

Supplementary Figure S3. Increased functional connectivity in the visual association network in female infants (Bonferroni-corrected). Brain regions showing increased functional connectivity within the visual association RSN in female infants. Example axial, sagittal and coronal slices for meaningful spatial patterns are shown, overlaid on a T1 structural template and displayed in radiological convention. T-statistic maps were thresholded at $p < 0.025/11$ (i.e. $p < 0.002$) (FWE corrected). White lines represent the outline of the group-ICA visual association network, thresholded at $Z > 3$.

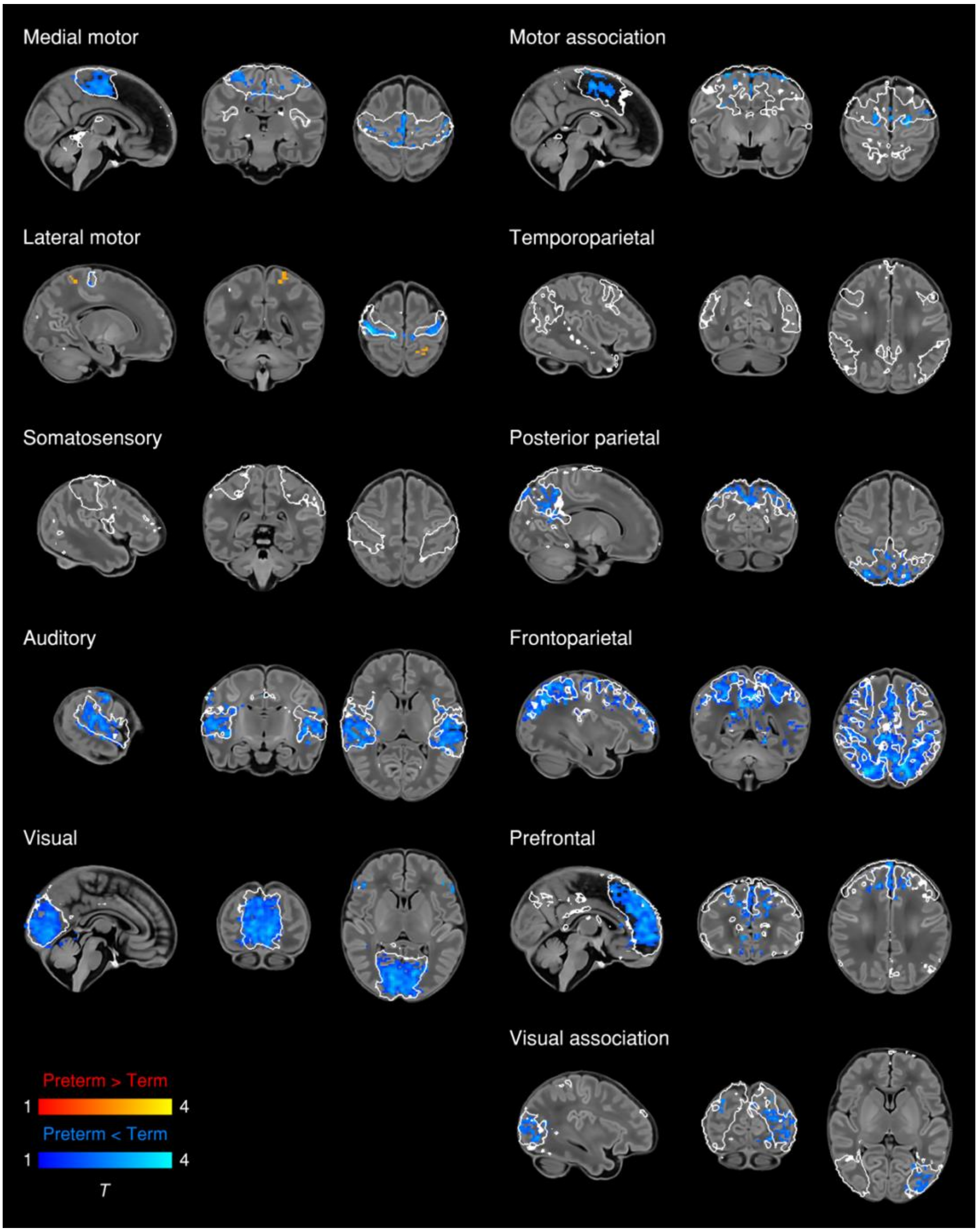

Supplementary Figure S4. Effect of preterm birth on functional connectivity (Bonferroni-corrected). Group differences in functional connectivity between term- and preterm-born infants scanned at 37-43.5 weeks PMA. Coloured t-statistic maps thresholded at $p < 0.025/11$ (i.e. $p < 0.002$) (FWE corrected) show brain regions with reduced (blue) or increased (red-yellow) connectivity in preterm-born infants. Example sagittal, coronal, and axial slices for meaningful spatial patterns within each RSN are shown, overlaid on a T1 structural template and displayed in radiological convention. White lines represent the outlines of the group-ICA RSNs, thresholded at $Z > 3$

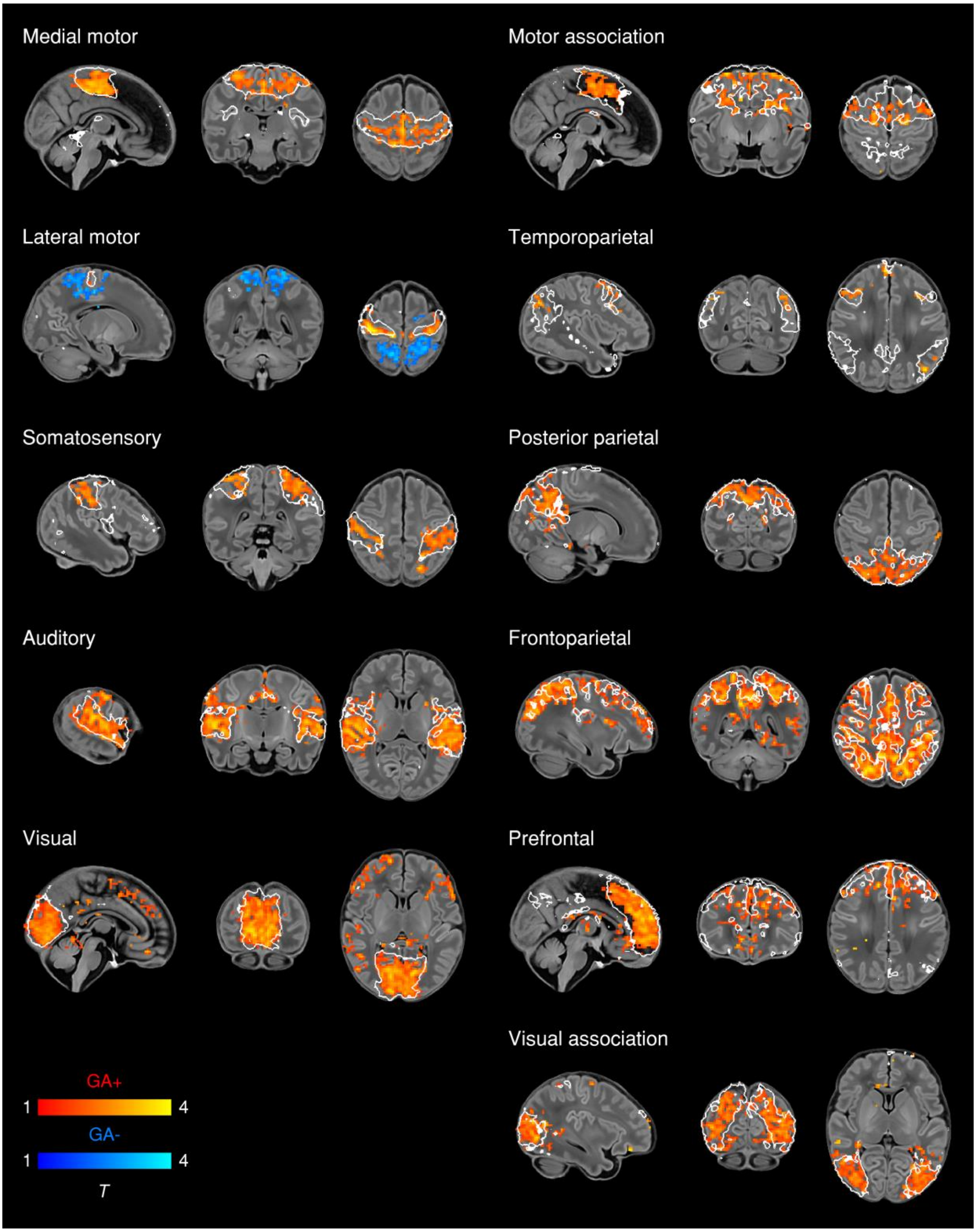

Supplementary Figure S5. Effect of gestational age at birth on functional connectivity. Association of functional connectivity and gestational age at birth (GA) in term- and preterm-born infants scanned at 37-43.5 weeks PMA. Coloured t-statistic maps thresholded at $p < 0.025$ (FWE corrected) show connectivity in brain regions negatively (blue) or positively (red-yellow) associated with gestational age at birth. Example sagittal, coronal, and axial slices for meaningful spatial patterns within each RSN are shown, overlaid on a T1 structural template and displayed in radiological convention. White lines represent the outlines of the group-ICA RSNs, thresholded at $Z > 3$.

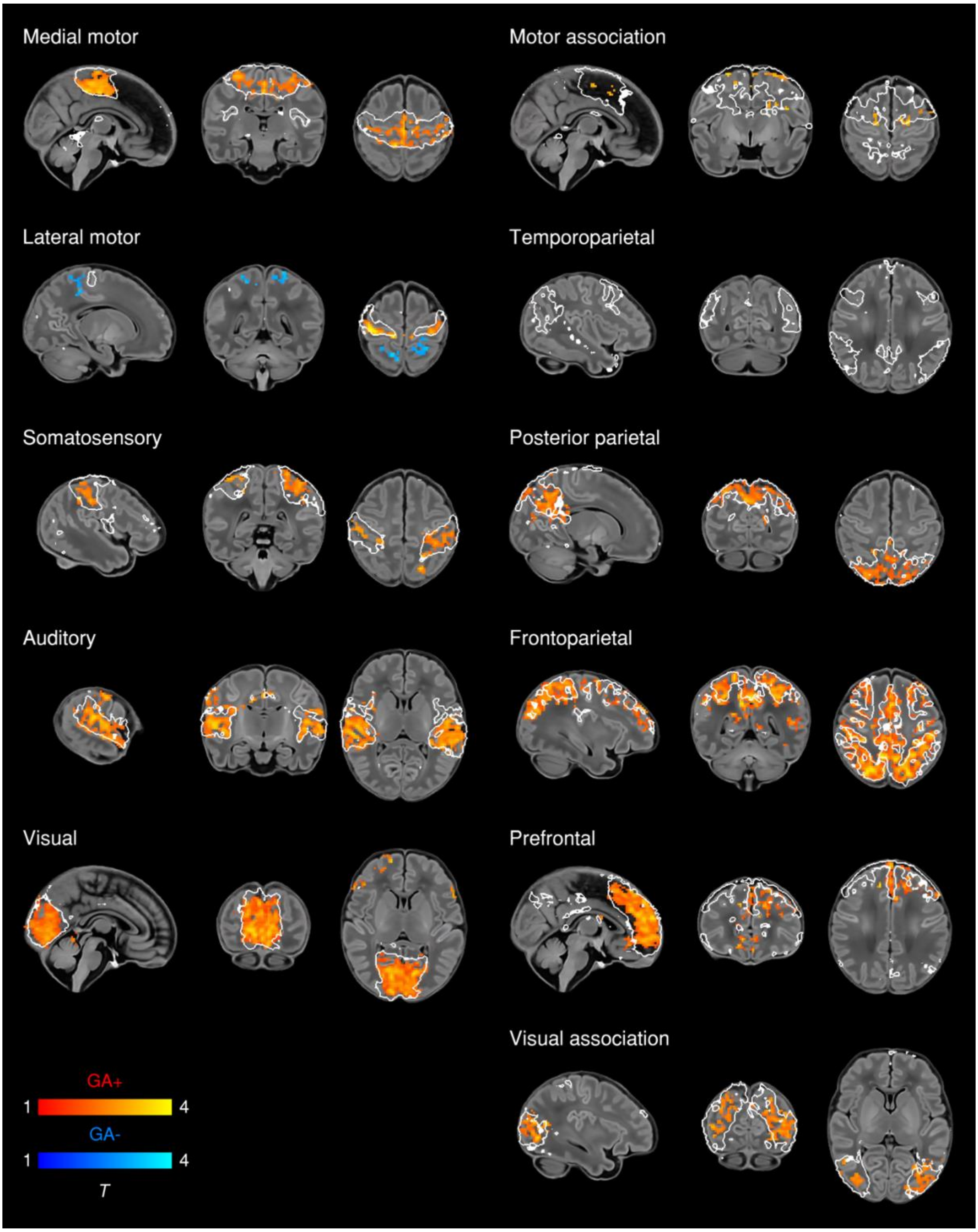

Supplementary Figure S6. Effect of gestational age at birth on functional connectivity (Bonferroni-corrected). Association of functional connectivity and gestational age at birth (GA) in term- and preterm-born infants scanned at 37-43.5 weeks PMA. Coloured t-statistic maps thresholded at $p < 0.025/11$ (i.e. $p < 0.002$) (FWE corrected) show connectivity in brain regions negatively (blue) or positively (red-yellow) associated with gestational age at birth. Example sagittal, coronal, and axial slices for meaningful spatial patterns within each RSN are shown, overlaid on a T1 structural template and displayed in radiological convention. White lines represent the outlines of the group-ICA RSNs, thresholded at $Z > 3$.

Supplementary Table S1. Voxels in Fig. 2 significant at $p < 0.025$ were localised to the AAL atlas, applied in the 40-week standard space.

Posterior parietal RSN

AAL region of interest	GM	Deep GM	Cerebellum	WM	Other (CSF, skull)
8 Frontal_Mid_R	24	0	0	29	0
12 Frontal_Inf_Oper_R	0	0	0	1	0
14 Frontal_Inf_Tri_R	2	0	0	35	0
30 Insula_R	0	0	0	7	0
34 Cingulum_Mid_R	0	0	0	5	0
49 Occipital_Sup_L	0	0	0	1	0
58 Postcentral_R	0	0	0	5	0
61 Parietal_Inf_L	11	0	0	13	0
62 Parietal_Inf_R	1	0	0	13	0
63 SupraMarginal_L	28	0	0	11	6
64 SupraMarginal_R	0	0	0	7	0
65 Angular_L	4	0	0	1	0
68 Precuneus_R	0	0	0	20	0
No ROI	4	2	0	392	58
	74	2	0	540	64

Frontoparietal RSN

AAL region of interest	GM	Deep GM	Cerebellum	WM	Other (CSF, skull)
72 Caudate_R	0	1	0	0	0
No ROI	0	0	0	2	1
	0	1	0	2	1

Prefrontal RSN

AAL region of interest	GM	Deep GM	Cerebellum	WM	Other (CSF, skull)
3 Frontal_Sup_L	0	0	0	29	0
4 Frontal_Sup_R	0	0	0	6	0
7 Frontal_Mid_L	0	0	0	21	0
8 Frontal_Mid_R	6	0	0	7	0
10 Frontal_Mid_Orb_R	1	0	0	0	0
14 Frontal_Inf_Tri_R	0	0	0	1	0
23 Frontal_Sup_Medial_L	0	0	0	18	0
30 Insula_R	0	0	0	3	0
31 Cingulum_Ant_L	4	0	0	8	0
32 Cingulum_Ant_R	0	0	0	1	0
66 Angular_R	9	0	0	2	0
No ROI	0	9	0	393	74
	20	9	0	489	74

Visual association RSN

AAL region of interest	GM	Deep GM	Cerebellum	WM	Other (CSF, skull)
2 Precentral_R	26	0	0	13	0
3 Frontal_Sup_L	8	0	0	4	0
7 Frontal_Mid_L	13	0	0	3	0
43 Calcarine_L	13	0	0	11	1
44 Calcarine_R	42	0	0	19	4
45 Cuneus_L	37	0	0	21	0
46 Cuneus_R	53	0	0	29	6
47 Lingual_L	1	0	0	20	0
48 Lingual_R	17	0	0	4	2
49 Occipital_Sup_L	67	0	0	114	6

50 Occipital_Sup_R	40	0	0	95	6
51 Occipital_Mid_L	150	0	0	160	3
52 Occipital_Mid_R	51	0	0	85	0
53 Occipital_Inf_L	36	0	0	38	0
54 Occipital_Inf_R	0	0	0	1	0
55 Fusiform_L	18	0	0	14	0
56 Fusiform_R	2	0	0	4	1
58 Postcentral_R	2	0	0	2	0
59 Parietal_Sup_L	13	0	0	0	8
63 SupraMarginal_L	0	0	0	1	0
64 SupraMarginal_R	4	0	0	7	0
65 Angular_L	11	0	0	11	0
66 Angular_R	4	0	0	11	0
67 Precuneus_L	12	0	0	14	7
68 Precuneus_R	16	0	0	7	0
72 Caudate_R	0	1	0	0	0
80 Heschl_R	1	0	0	0	0
81 Temporal_Sup_L	5	0	0	23	0
82 Temporal_Sup_R	1	0	0	10	0
85 Temporal_Mid_L	89	0	0	61	0
86 Temporal_Mid_R	4	0	0	27	0
90 Temporal_Inf_R	0	0	0	6	0
Cerebellum	0	0	19	0	0
No ROI	24	4	3	253	295
	760	5	22	1068	339
